# Supplementary material for: A Critical View of the Application of the APEX Software (Aqueous Photochemistry of Environmentally-Occurring Xenobiotics) to Predict Photoreaction Kinetics in Surface Freshwaters
Source: Molecules. 2019 Dec 18;25(1):9. doi: 10.3390/molecules25010009 (PMC7017383; doi:10.3390/molecules25010009)
Supplement: Supplementary file 1 [file molecules-25-00009-s001.zip › 3.2.4_gcc-4.4.0.zip!/3.2.4_gcc-4.4.0/3.2.4_gcc-4.4.0/doc/octave/html/interpreter/Statements.html]

Statements - Untitled


Next: Functions and Scripts,
Previous: Evaluation,
Up: Top


---

## 10 Statements

Statements may be a simple constant expression or a complicated list of
nested loops and conditional statements.

Control statements such as `if`, `while`, and so on
control the flow of execution in Octave programs. All the control
statements start with special keywords such as `if` and
`while`, to distinguish them from simple expressions.
Many control statements contain other statements; for example, the
`if` statement contains another statement which may or may not be
executed.

Each control statement has a corresponding end statement that
marks the end of the control statement. For example, the
keyword `endif` marks the end of an `if` statement, and
`endwhile` marks the end of a `while` statement. You can use
the keyword `end` anywhere a more specific end keyword is expected,
but using the more specific keywords is preferred because if you use
them, Octave is able to provide better diagnostics for mismatched or
missing end tokens.

The list of statements contained between keywords like `if` or
`while` and the corresponding end statement is called the
body of a control statement.

- The `if` Statement- The `switch` Statement- The `while` Statement- The `do-until` Statement- The `for` Statement- The `break` Statement- The `continue` Statement- The `unwind_protect` Statement- The `try` Statement- Continuation Lines
